# Mapping species richness using opportunistic samples: a case study on ground-floor bryophyte species richness in the Belgian province of Limburg

**DOI:** 10.1038/s41598-019-55593-x

**Published:** 2019-12-13

**Authors:** Thomas Neyens, Peter J. Diggle, Christel Faes, Natalie Beenaerts, Tom Artois, Emanuele Giorgi

**Affiliations:** 1 Centre for Statistics, Data Science Institute, Hasselt University, Agoralaan, Building D, B-3590 Diepenbeek, Belgium; 2 Centre for Environmental Sciences, Faculty of Sciences, Hasselt University, Agoralaan, Building D, B-3590 Diepenbeek, Belgium; 3 Leuven Biostatistics and statistical Bioinformatics Centre, Faculty of Medicine, KU Leuven, Kapucijnenvoer 35, block D, box 7001, B-3000 Leuven, Belgium; 4 Centre for Health Informatics, Computing, and Statistics, Lancaster Medical School, Lancaster University, Lancaster, LA1 4YW United Kingdom

**Keywords:** Biodiversity, Biodiversity, Ecological modelling, Statistics

## Abstract

In species richness studies, citizen-science surveys where participants make individual decisions regarding sampling strategies provide a cost-effective approach to collect a large amount of data. However, it is unclear to what extent the bias inherent to opportunistically collected samples may invalidate our inferences. Here, we compare spatial predictions of forest ground-floor bryophyte species richness in Limburg (Belgium), based on crowd- and expert-sourced data, where the latter are collected by adhering to a rigorous geographical randomisation and data collection protocol. We develop a log-Gaussian Cox process model to analyse the opportunistic sampling process of the crowd-sourced data and assess its sampling bias. We then fit two geostatistical Poisson models to both data-sets and compare the parameter estimates and species richness predictions. We find that the citizens had a higher propensity for locations that were close to their homes and environmentally more valuable. The estimated effects of ecological predictors and spatial species richness predictions differ strongly between the two geostatistical models. Unknown inconsistencies in the sampling process, such as unreported observer’s effort, and the lack of a hypothesis-driven study protocol can lead to the occurrence of multiple sources of sampling bias, making it difficult, if not impossible, to provide reliable inferences.

## Introduction

Citizen science is a body of research in which scientific investigations are carried out through the involvement of volunteers from the general public in collaboration with experts. This has received increased attention in different scientific fields over the last decade, including ecology^1–4^ where public participation provides a low-cost method for data collection in order to provide timely information on ecological processes. For example, in ornithology, studies on the distribution of British birds have used citizen-science data (CSD) to record breeding locations^5^ and ringing data, partly collected by citizens, have been used to investigate autumn migration in the French-Swiss Alps^6^. CSD have also been used to assess the effects of habitat degradation on avian population dynamics^7^, invertebrate distributions^8^, and habitat use of rare species^9^.

Price & Lee^10^ define a set of citizen science categories, based on varying degrees of citizens’ involvement and adherence to a pre-arranged study protocol. When we refer to CSD in this study, we focus on the *active contributory model*, in which participants actively engage in data collection and/or processing, while making individual decisions regarding sampling strategies. Although this model is often used in wildlife monitoring, its economical benefits are offset by the absence of a consistent study design methodology. As a result, it is inadvisable to use CSD for modelling complex ecological processes without understanding the mechanisms that might yield biased inferences on the phenomenon under investigation^11^. Isaac *et al*.^12^ identify four main sources of bias in this type of CSD: (i) temporal and (ii) spatial unbalance in data collection, (iii) uneven species detectability, and (iv) uneven sampling effort per visit. Although (i), (ii), and (iii) are not exclusive to CSD, they are likely to be exacerbated in the absence of predefined sampling guidelines. Uneven sampling effort (iv) is more particular to CSD, and may be the product of different sampling inconsistencies, such as differences in the durations spent on the field and the number of data collectors per visit. Hadj-Hammou *et al*.^13^ argue that the development of methods to investigate the drivers behind the behaviour of citizens is essential to address these issues.

Recent studies on the biodiversity of ecosystems have reported mixed results on the usefulness of CSD. For example, variation in observational skills among citizens has been found to be an important source of bias in detecting adelgids^14^ and anurans^15^. In contrast, Delaney *et al*.^16^ concluded that CSD provided useful insights into the distribution of native and invasive crabs, but also highlighted that age and university training of the collector were associated with identification skills. Boudreau & Yan^17^ found that citizens were able accurately to detect invasions of non-native water fleas in Canadian lakes. van Strien *et al*.^18^ found a good match between predicted trends from CSD and monitoring data on the distribution of dragonflies and butterflies.

Species richness is defined as the number of species present in an ecosystem and is used as a measure of biodiversity. Studies on species richness frequently rely on CSD but their opportunistic nature is often ignored or is addressed through the use of overly simplistic approaches that cannot account for specific sources of sampling bias. An exception is given by Carota *et al*.^19^, who model species richness from historical data using a semi-parametric Poisson model with random effects drawn from a mixture of Dirichlet processes. They show that this data-driven approach better captures the large heterogeneity induced by the opportunistic nature of the underlying sampling mechanism than standard Poisson mixed models. Over the last decade, research based on the use of CSD has been mainly focused on the modelling of geographical distributions of a single animal species. A commonly used approach consists of subsampling the data based on the number of location visits and the amount of species collected per location. For example, in their analysis of European ladybird declines, Roy *et al*.^20^ only consider data collected over a 1 × 1 km^2^ regular grid, where at least two common species were observed. Similarly, Kuussaari *et al*.^21^ discard all data on a 10 × 10 km^2^ regular grid with less than 40 records of focal farmland butterfly species reported before 1960 to assess changes in their spatial distribution, resulting in the loss of 85% of data points from cells visited before 1960. A generally more robust approach, which we also follow in this paper, is to account for potential sources of bias through the inclusion of key explanatory variables to the model. For example, Szabo *et al*.^22^ include the total number of species to model the abundance of several avian species as a proxy for the observer’s effort. Kelling *et al*.^23^ and Johnston *et al*.^24^ use model-based predictions of the number of recorded species to correct for observer-specific species’ detectability skills. Occupancy-detection models, originally proposed to model imperfect detection of species^25,26^, have also been used to account for observation and reporting bias^18^. However, these models require presence/absence information from repeated site visits and often result in an excessive aggregation of the data over time and space, which is questionable when locations are revisited at highly irregular time intervals.

In this paper, we focus on the spatial prediction of species richness of ground-floor bryophytes in Limburg, Belgium, using data from an expert source and CSD. More specifically, the first source consists of randomised survey data (RSD) collected by a biologist who adheres to a predefined and randomised sampling design protocol. The CSD, in contrast, include non-randomised opportunistic samples obtained by a team of two individuals who are occasionally joined by other collectors. An important aspect of our approach is the explicit modeling of the spatial correlation between observations. This issue has been extensively addressed in modelling species distributions^27–29^ with some attempts to account for sampling and detection bias (see, for example, Pacifici *et al*.^30^). Conn *et al*.^31^ use geostatistical methods to model ecological data in the presence of *preferential sampling*. This term refers to a special case of opportunistic sampling in which there is stochastic dependence between the sampling design and the reported species counts^32^. In the context of species richness modelling, Chakraborty *et al*.^33^ use spatial point processes to estimate the distribution of six different species separately. From each individual point process model, they then draw posterior samples for the estimated intensity functions to predict the overall species richness.

The objective of our study is to assess the reliability of the CSD in predicting bryophyte richness. Hence, we first use a log-Gaussian Cox process model to understand what variables might have affected the opportunistic nature of the sampling mechanism in the CSD. We then fit geostatistical Poisson models to the two data-sets and compare the resulting spatial estimates for bryophyte richness. Model-based geostatistics^34^ provides a principled, likelihood-based approach to inference. It also exploits the correlation between recordings of species richness by accounting for unmeasured environmental factors through the inclusion of a latent spatial Gaussian process in the linear predictor of the response variable. To the best of our knowledge, this is the first study that, unlike others where groups of citizens are large and heterogeneous, (i) uses a model-based approach to describe the sampling pattern of individual collectors in a CSD context and (ii) validates the use of the CSD for mapping species richness using geostatistical methods.

## Methods

### Data

Our study area is the province of Limburg, covering 2,414 km^2^ in the Eastern part of Belgium; we do not include Voeren, a smaller exclave of the Limburg province. The data, which are publicly available^35^, are obtained from two sources: the RSD correspond to observations recorded by a biologist working for the Belgian Nature and Forest Agency (ANB); the CSD consist of opportunistic samples collected by two citizens for the Umbrella for Nature Research in Limburg (LIKONA). The citizens lived in the western part of Limburg and were occasionally joined by other citizens. The outcome of interest, available from both sources, is the number of distinct forest ground-floor bryophyte species determined in a lab through microscopy detection or visual inspection of a moss sample. Species richness is often used as an index of biodiversity when the total counts per species are difficult or impossible to assess^36^. Both data-sets include information on the date of collection, the name of the data collectors and the GPS locations of the sampled cell from a 1 by 1 km regular grid created by the Institute for Floristics in Belgium and Luxembourg (IFBL). Each data collector reported all bryophyte species observed during the surveys.

**Figure 1:**
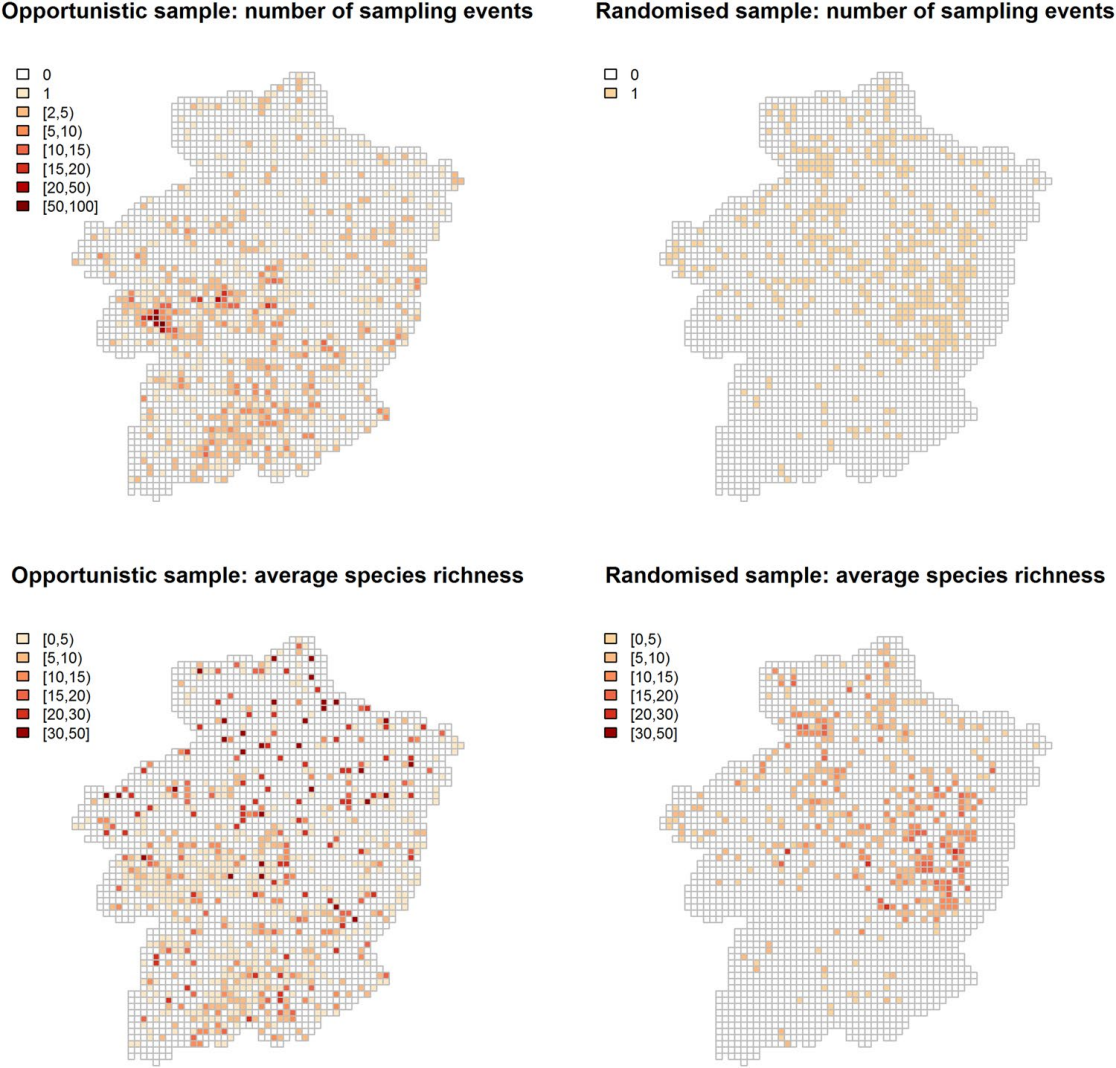
Maps of the number of sampling events and the average observed forest ground-floor species richness over a 1 by 1 km regular grid, from the two data sources.

Geographical summaries of sampling events and the observed species richness for both the RSD and CSD are given in Fig. 1. The RSD were collected between 1997 and 1999 and include a total of 420 locations in Limburg that were selected using a randomised systematic lattice-based sampling design, which was restricted to the forested areas of Limburg; a 1 km × 1 km raster was randomly superimposed on the map of Limburg. All intersections between horizontal and vertical raster lines that landed on an area that was officially defined as a forest, were designated as sampling locations. During the sampling, the data collector walked no more than 50 m in one direction, starting from the sampling location, and repeated this in directions at 90°, 180°, and 270° angles from the initial direction. Sampling was restricted to the prescribed trajectory. The collector assembled samples of all ground-floor bryophytes that, by visual inspection, were species not yet encountered during the sampling event. Those samples were later investigated under laboratory circumstances. There was no fixed or maximal duration of a sampling event, which is a sensible choice, since data gathering is likely to take a longer time on more biodiverse trajectories. More details can be found in Afdeling Bos & Groen^37^. Figure 1 (top right panel) shows the resulting set of sampled locations. The CSD contain 2,088 recordings of bryophyte richness from opportunistically selected locations between 1985 and 2009 (Fig. 1, top left panel). From interviews, we learnt that the selection of locations was often based on their proximity to the home of the two main collectors, with preference for those locations that were more biodiverse according to the collectors’ knowledge. Except for a number of sites relatively close to their homes, locations were rarely revisited. The goal of the data collection carried out by the two citizens was to cover the largest possible area in the Limburg province. As a result, unlike the RSD, sampling was not restricted to forested areas and the citizens reported observations of all possible bryophyte species, regardless of habitat and substrate. The two main CSD collectors were volunteer bryophyte experts, with species identification skills that can be assumed to be similar to the RSD’s expert knowledge. The observers’ efforts, such as the time spent in the field, varied greatly between events and were not documented. However, the interviews also revealed that both collectors were mostly in close proximity and followed the same trajectory when collecting data. There is no available documentation of all Belgian forest ground-floor bryophytes. The species labelled here as forest ground-floor bryophytes, occur in forests, but are not necessarily restricted to forested areas and ground floors, which means that the citizens could have found these species at locations other than forests and/or ground floors. Furthermore, since spatial information is only available on the grid-level, which mostly consists of multiple habitat types, we do not know which of the CSD’s samples were taken from ground floors of forests. As a pragmatic solution, we extract from the CSD database only the observations of species that occur in the RSD data.

### Environmental variables

**Figure 2:**
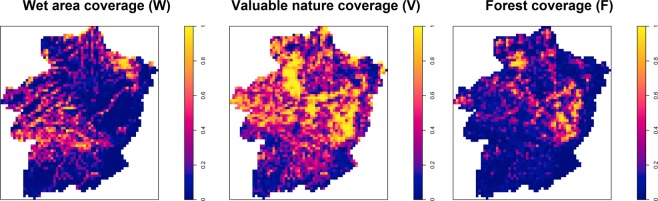
Relative coverages of wet areas, valuable nature, and forests, per IFBL cell.

We use information on wetness, environmental evaluation and forest cover to generate ecological variables that might help to explain the spatial variation in bryophyte species richness. The Flemish geological map^38^, last updated in 2017, provides wetness categories for each landscape zone within the study area. Since multiple landscape zones may fall within each IFBL cell, we compute the proportion of wet landscape zones (henceforth, *W*) as shown in Fig. 2. Similarly, we compute the relative coverage of valuable nature (henceforth *V*; Fig. 2), defined as the proportion of landscapes within an IFBL cell that are wholly or partly labelled as “very valuable” or “valuable” according to the Belgian Biological Evaluation Map anno 2016^39^. Nature evaluation is based on several correlated environmental characteristics, including habitat type, presence of ecologically important fauna and flora and the level of human disturbance. Hence, using the variable *V* allows us to avoid multicollinearity issues while capturing the main environmental features of each IFBL cell. We do not use temporally varying values for *W* and *V*, but it is reasonable to assume that these have only slightly changed between 1985 and 2009, since environmental planning in Limburg has undergone only minor changes throughout the last 40 years. Finally, the relative forest coverage within each IFBL cell (henceforth, *F*) was calculated, based on the Flemish Forest Map^40^.

### Statistical analysis

#### Modelling of opportunistic sampling as a preferential sampling scheme

Preferential sampling is a term coined by Diggle *et al*.^32^ to denote processes that generate sampling locations *X* that are stochastically dependent on the underlying spatial process of interest, which in our case corresponds to the species richness at any given location in Limburg. The opportunistic samples from the CSD study might in fact have been generated from a similar mechanism whereby the stochastic dependence between species richness and the selection of a location for data collection is induced by several unmeasured factors, which might increase the likelihood of finding more species. The RSD’s sampling locations were chosen non-preferentially, as they were an independent sample from a (non-uniform) distribution on the region of Limburg, in which locations outside forests had a sampling probability of zero, while locations inside forests had an equal sampling probability.

Ignoring stochastic dependence caused by preferential sampling can invalidate our predictive inferences on the outcome of interest, as shown in Diggle *et al*.^32^. Our modelling strategy is inspired by Pati *et al*.^41^ who extended the geostatistical method of Diggle *et al*.^32^ using a bivariate spatial process. They proposed a joint modelling approach where a geostatistical model of the outcome of interest shares a spatial random effect with a log-Gaussian Cox process (LGCP) that models the choice of sampling locations. Note that by definition, preferential sampling refers to stochastic dependence between species richness and location choice; in other words, it reflects unexplained variability that is shared between both models. Preferential sampling does not occur as a result of variability that is explained by shared predictors.

We proceed as follows. To understand how distance from home and evaluation of nature affected the choice of a sampling location, *x*, in the CSD data, we model the latter as the realisation of a LGCP with intensity

1 $${\Lambda }(x)=exp\{{\alpha }_{0}+{\alpha }_{1}D(x)+{\alpha }_{2}V(x)+S(x)\},$$

where: *D*(*x*) is the distance between *x* and the collectors’ residential municipality; *V*(*x*) is the evaluation of nature at location *x*; and *S*(*x*) is a stationary and isotropic Gaussian process with zero mean, variance *σ*^2^ and Matérn^42^ correlation function given by

$$\rho (u;\phi ,\kappa )={\{{2}^{\kappa -1}\varGamma (\kappa )\}}^{-1}{(\frac{u}{\phi })}^{\kappa }{K}_{\kappa }(\frac{u}{\phi }),$$

where: *u* > 0 is the Euclidean distance between any two locations; *ϕ* is a scale parameter regulating how quickly the spatial correlation decays to zero for increasing distance; and *K*_*k*_(.) denotes the modified Bessel function of the second kind, of order *κ* > 0. Zhang^43^ warns that under fixed-domain asymptotics *σ*, *ϕ*, and *κ*, cannot be estimated consistently; as a pragmatic approach, we therefore set *κ* = 1. Fitting the LGCP model is computationally intensive; we therefore approximate *S*(*x*) using a stochastic partial differential equations (SPDE) approach. SPDE uses a triangulation method based on Gaussian weights with Markov dependencies that approximate the Matérn covariance structure. More details can be found in Lindgren *et al*.^44^. The triangulation mesh was constructed using recommendations outlined in Krainski *et al*.^45^.

The objectives of the geostatistical analysis are to model species richness from the two data sources and to quantify the differences between the resulting predictive inferences. In the case of the CSD, we also account for bias that might be induced by preferential sampling.

Let *Y*_*j*_(*x*_*i*_, *t*_*i*_) denote the total number of forest ground-floor bryophyte species collected at location *x*_*i*_ and time *t*_*i*_  ∈ {1985, …, 2009} from source *j* ∈ {*CSD*, *RSD*}. Conditionally on a spatial Gaussian process *U*_*j*_(*x*) and Gaussian noise *Z*_*j*_(*x*_*i*_), we assume that *Y*_*j*_(*x*_*i*_, *t*_*i*_) are mutually independent Poisson random variables with means *μ*_*j*_(*x*_*i*_, *t*_*i*_). More specifically, for *j* = *CSD*, we write

2 $$\begin{array}{rcl}{\mu }_{CSD}({x}_{i},{t}_{i}) & = & exp\{{\beta }_{0,CSD}+{\beta }_{1,CSD}W({x}_{i})\\  &  & +{\beta }_{2,CSD}V({x}_{i})+{\beta }_{3,CSD}F({x}_{i})+{\beta }_{4}log({C}_{i})\\  &  & +f({t}_{i})+\gamma \hat{S}({x}_{i})+{U}_{CSD}({x}_{i})+{Z}_{CSD}({x}_{i})\}\end{array}$$

where: $\hat{S}({x}_{i})$ is the predictive mean of the spatial process in Eq. (1) at location *x*_*i*_ used to account for preferential sampling. Based on the results from our exploratory analysis (Appendix; Fig. A.1), we assume a log-linear relationship between *Y*_*j*_(*x*_*i*_, *t*_*i*_) and *Ŝ*(*x*_*i*_); *W*(*x*_*i*_), *V*(*x*_*i*_) and *F*(*x*_*i*_) are the three spatial variables, shown in Fig. 2, at location (*x*_*i*_); *C*_*i*_ is the number of collectors; and *f*(*t*_*i*_) is a cubic spline with knots at 1990, 1995, 2000, 2002, 2004, and 2006. Note that, if all factors that explain the spatial variation in the choice of the sampled locations in the CSD study were available, this would lead to *S*(*x*_*i*_) = 0 for all *i*, and hence $\hat{S}({x}_{i})$ = 0 meaning that bias arising from preferential sampling would be completely removed from the model for species richness.

Finally, for *j* = *RSD*, we write

3 $${\mu }_{RSD}({x}_{i},{t}_{i})=exp\{{\beta }_{0,RSD}+{\beta }_{1,RSD}W({x}_{i})+{\beta }_{2,RSD}V({x}_{i})+{\beta }_{3,RSD}F({x}_{i})+{\beta }_{5}{t}_{i}^{st}+{U}_{RSD}({x}_{i})+{Z}_{RSD}({x}_{i})\},$$

where: ${t}_{i}^{st}={t}_{i}-1997.$ We use ${\nu }_{j}^{2}$ and *ψ*_*j*_ to denote the variance and scale of the exponential spatial covariance functions used for *U*_*j*_(*x*).

#### Parameter estimation and predictive comparison

We estimate the parameters of the LGCP model and the Poisson geostatistical models in Section 2.3.1 using the Monte Carlo maximum likelihood (MCML) method^46^, implemented in the PrevMap package^47^. More technical details on how the SPDE approach, used for approximation of the Gaussian process *S*(*x*), and the MCML method are implemented in order to obtain the parameter estimates can be found in Chapter 7 of^48^. We wish to compare the resulting predictions for *μ*_*RSD*_(*x,t*) and *μ*_*CSD*_(*x,t*) at locations and times for which both sources provide enough information, noting that *μ*_*CSD*_(*x,t*) corresponds to the average species richness adjusted for preferential sampling bias in CSD. We then set *t* = 1998 and consider locations *x* such that the estimated spatial correlation between *U*_*j*_(*x*) and *U*_*j*_(*x*_*c*_) is no less than 0.75, with *x*_*c*_ denoting the sampled location from either of two sources that is closest to *x*, for *j* = *RSD*, *CSD*. We then summarise the discrepancy between *μ*_*RSD*_(*x*,*t*) and *μ*_*CSD*_(*x*,*t*) through their relative difference

$$RD(x)=\{{\hat{\mu }}_{CSD}(x,t)-{\hat{\mu }}_{RSD}(x,t)\}/max\{{\hat{\mu }}_{CSD}(x,t),{\hat{\mu }}_{RSD}(x,t)\}.$$

In computing ${\hat{\mu }}_{CSD}(x,t)$, the number of collectors is set to *C*_*i*_ = 2. As noted in Section 2.1, we assessed that the two collectors from the CSD acted as one in view of their strong interaction during the data collection.

## Results

**Figure 3:**
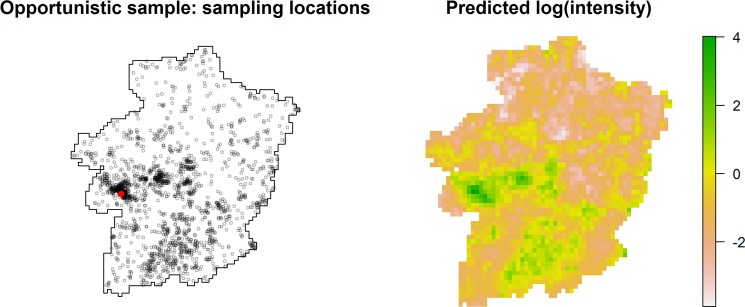
Opportunistic sample. Left: observed sampling locations, with random jitter and a red dot indicating the central point of the collectors’ residential municipality. Right: log-transformed intensities, predicted by the log-Gaussian Cox model.

The triangulation mesh used for the SPDE approximation in the LGCP model is provided in the Appendix (Fig. A.2). The parameter estimates in Table 1 indicate that the data collectors, on average, visited locations closer to home and of higher natural value more intensely. In particular, we find that evaluation of nature has the strongest effect, estimating that a location within an environmentally 100% valuable environment has a sampling intensity about (*exp*{2.778}≈) 16 times larger, with the other variables kept equal. However, the predicted surface from the LGCP model (Fig. 3) suggests the presence of additional factors affecting the two collectors’ choice of locations. The map shows that a large swathe in the south of Limburg, both far from the collectors’ homes and of low natural value, was also sampled with moderate intensity.

**Table 1:** Results for the LGCP model for the opportunistic sampling process of the CSD.

	Parameter	estimate	95% C.I.
intercept	*α*_0_	2.970	[2.822;3.117]
distance to house	*α*_1_	−0.031	[−0.035;−0.027]
value of nature	*α*_2_	2.778	[2.601;2.955]
spatial variance	log(σ^2^)	2.487	[2.302;2.673]
scale	log(*ϕ*)	2.678	[2.176;3.180]

**Figure 4:**
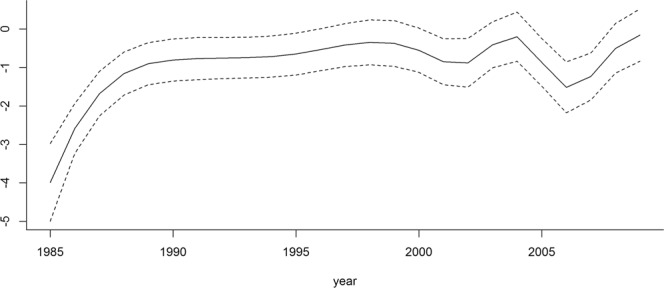
Fitted spline effect (solid line) in the CSD geostatistical model, along with 95% confidence intervals (dashed lines).

**Figure 5:**
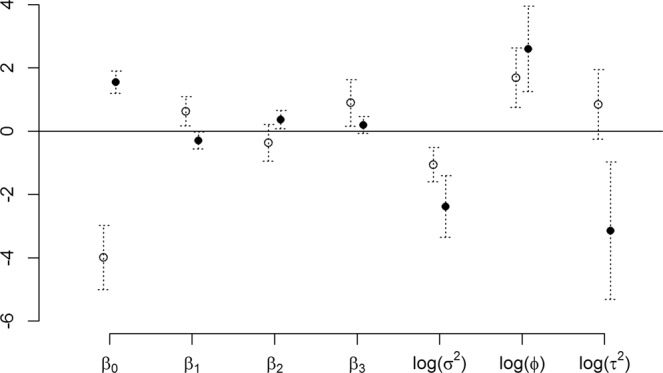
Visual comparison of the point estimates (open circles for the citizen science data (CSD); solid circles for the random study data (RSD)) and 95% confidence intervals (dotted lines) for the parameters common to two geostatistical models that were fitted to the CSD and RSD.

For the CSD, we did not find evidence of an association between the predictive mean of the residual spatial process *S*(*x*) and species richness, as indicated by the non-significant estimate for the parameter *γ* at the conventional 5% level ($\hat{\gamma }=-\,0.031$, 95% confidence interval [−0.106; 0.044]). As expected, the mean level of species richness significantly increases as the number of collectors also increases ($\widehat{{\beta }_{4}}=1.905$, 95% confidence interval [1.513; 2.396]). The spline function fitted in the CSD model is shown in Fig. 4. The time effect is almost flat during the years in which the RSD were collected (1997 to 1999). For the RSD, the linear time effect was negative ($\widehat{{\beta }_{5}}=-\,0.083$, 95% confidence interval [−0.138; −0.028]). Table 2 and Fig. 5 provide comparisons for the estimates of parameters used in the geostatistical models for both CSD and RSD. We found contrasting results. In the CSD, the estimated regression coefficients for wetness and forest cover were both positive, while the effect of evaluation of nature was not significant. For RSD, these three effects were significantly negative, non-significant and significantly positive, respectively.

**Table 2:** Estimates and 95% confidence intervals of parameters common to the CSD and RSD geostatistical models for species richness.

	parameter	estimate	95% C.I.	estimate	95% C.I.
		CSD		RSD	
intercept	*β*_0_	−3.990	[−5.003;−2.978]	1.541	[1.189;1.894]
wetness	*β*_1_	0.627	[0.166;1.089]	−0.295	[−0.566;−0.025]
value of nature	*β*_2_	−0.365	[−0.948;0.217]	0.366	[0.078;0.653]
forest cover	*β*_3_	0.984	[0.161;1.627]	0.193	[−0.074;0.461]
spatial variance	log(*σ*^2^)	−1.060	[−1.602;−0.518]	−2.379	[−3.354;−1.404]
scale	log(*ϕ*)	1.686	[0.747;2.625]	2.596	[1.245;3.948]
nugget	log(*τ*^2^)	0.845	[−0.256;1.946]	−3.146	[−5.318;−0.975]

Comments on the temporal trends, and the effects of group size in the CSD analysis, not reported in this table, are provided in the main text.

**Figure 6:**
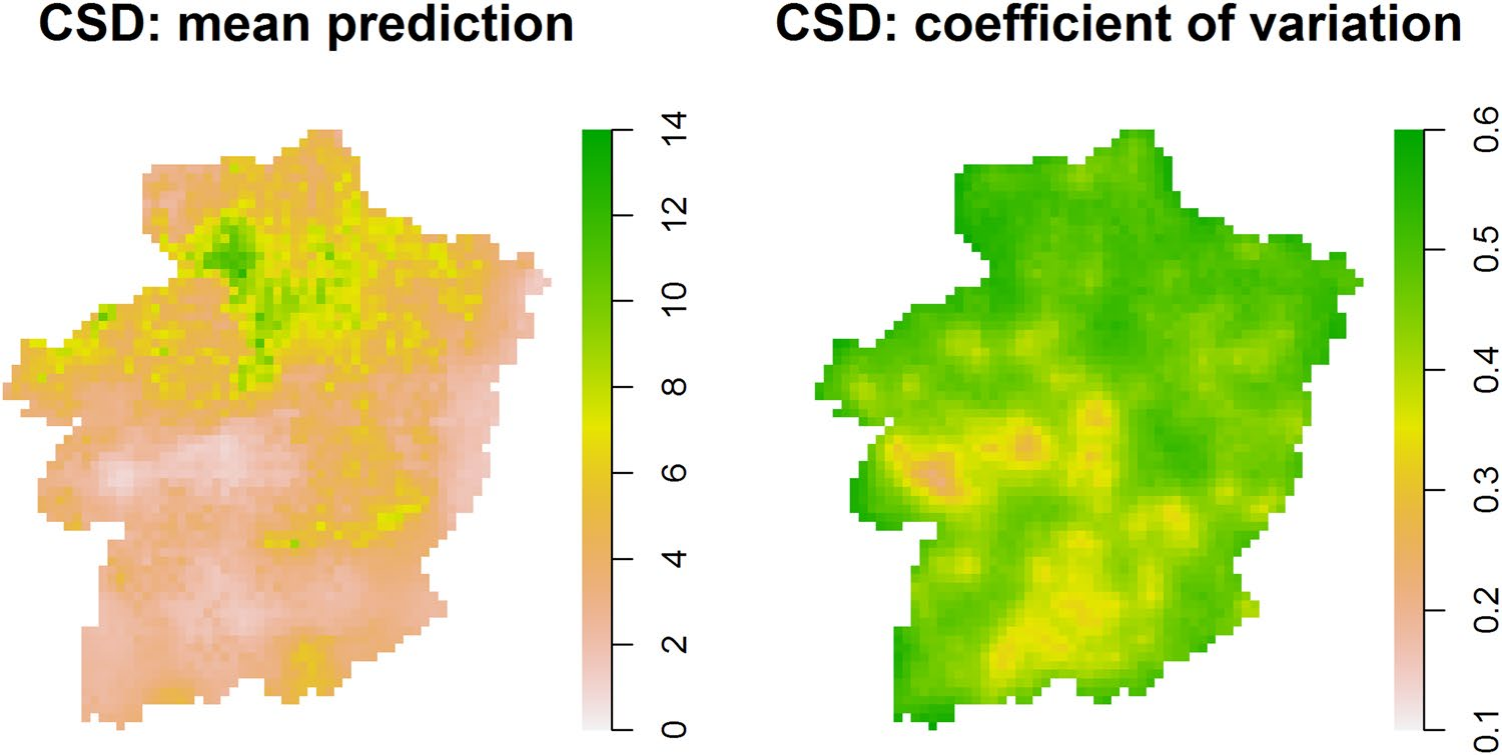
Mean forest ground-floor bryophyte species richness prediction and coefficient of variation (standard error/mean prediction) for 1998 and 2 data collectors, based on the CSD.

**Figure 7:**
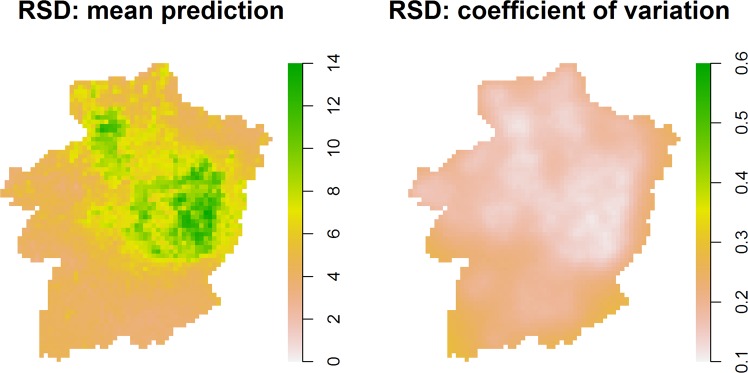
Mean forest ground-floor bryophyte species richness prediction and coefficient of variation (standard error/mean prediction) for 1998, based on the RSD.

**Figure 8:**
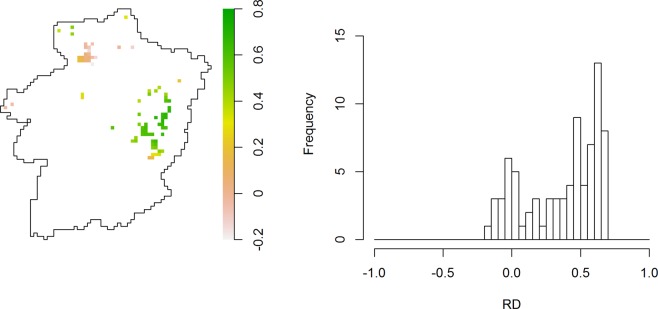
A map and histogram of relative differences (*RD*(*x*)) between mean predictions for 1998, based on CSD ad RSD, for locations that were visited in both data collections.

Figures 6 and 7 show the predicted spatial surfaces for ${\hat{\mu }}_{CSD}(x,t)$ and ${\hat{\mu }}_{RSD}(x,t)$ for *t* = 1998. Predictions from the CSD show higher values in species richness in the northern part of Limburg, while the RSD predictions point to increased values in the north-western and central-eastern parts of Limburg. In addition, Figures 6 and 7 show increased estimation error in the CSD-based predictions. Fig. 8 shows the relative difference at locations where both CSD and RSD could be assumed to be informative (see Section 2.3.2 for more details). We observe that overall the CSD provide lower predictions of species richness than the RSD, with relative differences ranging between −0.15 and 0.75.

## Discussion

We have assessed the reliability of opportunistic samples on forest ground-floor bryophyte species richness collected by two citizens in Limburg, Belgium, using data from an expert source who adheres to a randomised sampling regime as the gold-standard. In the first part of our analysis, we have fitted a log-Gaussian Cox process (LGCP) model to investigate spatial factors that might have affected the citizens’ choice of sampling locations. Our results indicate that locations with a higher value of nature and those located closer to the residential location of the collectors were sampled more intensely than others. However, the fitted LGCP model also suggests that there may be additional factors driving the sampling behaviour of the two citizens. These might be related to personal interests in a specific species or taxonomic group, as documented by Boakes *et al*.^49^. Other authors have highlighted that environmental features that facilitate monitoring often play an important role in citizens’ sampling choices. Hadj-Hammou *et al*.^13^ observed that citizens visited sampling locations with habitat types that are easily accessible and abundant throughout the study region more frequently than other locations. Ease-of-access has also been shown to be important in the opportunistic placement of camera traps^50^.

In the second part of the analysis, we have carried out spatial prediction of bryophyte species richness by fitting geostatistical Poisson models. For the citizen-science data, or CSD, we have accounted for potential bias induced by preferential sampling^32^ through the inclusion of the predictive mean of the spatial Gaussian process from the LGCP model into the linear predictor for species richness. This allows us to account for spatial factors that affect both the likelihood of a location being sampled and the variation in species richness. However, since this did not show any significant effect, we conclude that, in this case, preferential sampling may not capture all the main features of the opportunistic sampling process in the CSD. Other sources of bias might not be spatially structured and can only be accounted for when they are accurately reported by the data collector, which is not the case in our study. Here, group size was the only available variable that could be used as proxy for the heterogeneity in the collectors’ effort and it was found to have a significant effect on species richness in the CSD. In the CSD analysis, we found positive effects of wetness and forest cover. In contrast, the results from the randomised study (RSD) indicated a negative effect of the wetness index and a positive effect of environmental value. Finally, the spatial estimates for species richness differed greatly at many locations between the CSD and RSD, especially in the eastern parts of Limburg. Note that the estimates were generally smaller in the CSD than in the RSD.

Following the classification by Isaac *et al*.^12^ of the sources of bias in CSD, we draw the following conclusions. The spatial unbalance of the data was not an issue in our analysis, since our approach, unlike, for example, occupancy-detection models, does not require data to be aggregated over space. We account for uneven sampling effort by including group size as a covariate in the geostatistical model. However, we were not able to account for additional likely sources of uneven sampling effort as these were not reported. Examples of these are the time spent in the field and the trajectory covered by the citizens, two sampling parameters that are instead controlled for in the RSD’s study design. Another limitation of our approach is that it does not account for species- and observer-specific detectability. To account for species-specific detectability, we would require accurate prior information on the probability of detection and how this varies in space for each species so as to incorporate it into a model for the presence/absence of each species. Dorazio and Royle^51^ and Dorazio *et al*.^52^ predict species richness based on joint species-specific site-occupancy models, in which a species’ site-occupancy is modelled as a mixture of a Bernoulli process that determines the likelihood of the species’ presence or absence at a given location and a second Bernoulli process regulating how likely that species is to be observed at that location given its presence. The method requires a well-structured study protocol with site re-visits, both of which were unavailable for the CSD. Since only two collectors, occasionally joined by other citizens, carried out the collection of the CSD, variation in the observer-specific detectability skills is less problematic in our case. This is known to cause inferential issues when data are collected by a highly heterogeneous group of citizens^24^. Furthermore, in our study, collectors in both the CSD and RSD had extensive knowledge of bryophyte systematics and we therefore assume that they all have similar detectability skills. This is arguably a strong assumption, since observer-specific detectability has been shown to vary considerably, even among the most skilled data collectors^53^.

Most of the challenges in the analysis of CSD, especially in the active contributory model^10^ arise from the lack of a well-defined scientific hypothesis that might provide guidance for the sampling design. The underestimation in bryophyte species richness reported in our analysis of the CSD may be due to the fact that the two investigators were less likely to engage in the close inspection of species on the forest floors, which was the main focus of the RSD study. However, this can as well be an artefact of using the set of species that were collected in the RSD study to extract forest ground-floor bryophytes from the CSD data, which implied that forest ground-floor bryophyte species that were identified by the citizens, but not in the expert study, were not considered for the analysis.

A secondary issue in our study is that the resolution in which the spatial and/or temporal variation is recorded does not align with the resolution at which the true ecological process is at play; e.g., spatial trends in moss species richness will probably vary considerably within each 1km^2^ grid cell, but we are unable to investigate this. We face this problem in both CSD and RSD analyses, but it generally poses a difficulty in historical CSD surveys, which have become popular sources to investigate long-term ecological trends. This spatial misalignment can be one of the reasons why an ecological covariate such as value of nature was found to contribute less to the variability in the CSD outcome than expected.

We conclude that in general, we cannot trust inferences that are drawn purely from CSD that were collected without adherence to a strict sampling protocol, due to multiple unreported sources of sampling bias. These may be difficult, if not impossible, to account for in absence of detailed information on the sampling procedures adopted by the citizens. This does not imply that CSD within the active contributory model cannot provide useful information for estimating biodiversity, but rather that standard modelling approaches will be prone to failure. A more promising approach would be to combine the imperfect information from CSD with gold-standard data that can deliver unbiased spatial estimates. This has been achieved in the context of disease mapping^54,55^ where joint geostatistical models have been developed in order to remove bias from opportunistically collected samples by analyzing these jointly with data from randomised prevalence surveys. However, we could not apply this modelling framework in our study due to the relatively small temporal overlap between CSD and RSD, which does not allow to reliably estimate the spatially varying bias of the CSD.

## Supplementary information

Appendix

## Data Availability

The data are available at 10.5061/dryad.brv15dv5r.
