# Supplementary material for: Mapping species richness using opportunistic samples: a case study on ground-floor bryophyte species richness in the Belgian province of Limburg
Source: Sci Rep. 2019 Dec 13;9:19122. doi: 10.1038/s41598-019-55593-x (PMC6911062; doi:10.1038/s41598-019-55593-x)
Supplement: Supplementary file 1 — Appendix [file 41598_2019_55593_MOESM1_ESM.docx]

**Supplementary Material**

**Mapping species richness using opportunistic samples: a case study on ground-floor bryophyte species richness in the Belgian province of Limburg**

Thomas Neyens, Peter J. Diggle, Christel Faes, Natalie Beenaerts, Tom Artois, Emanuele Giorgi

**Appendix**

**
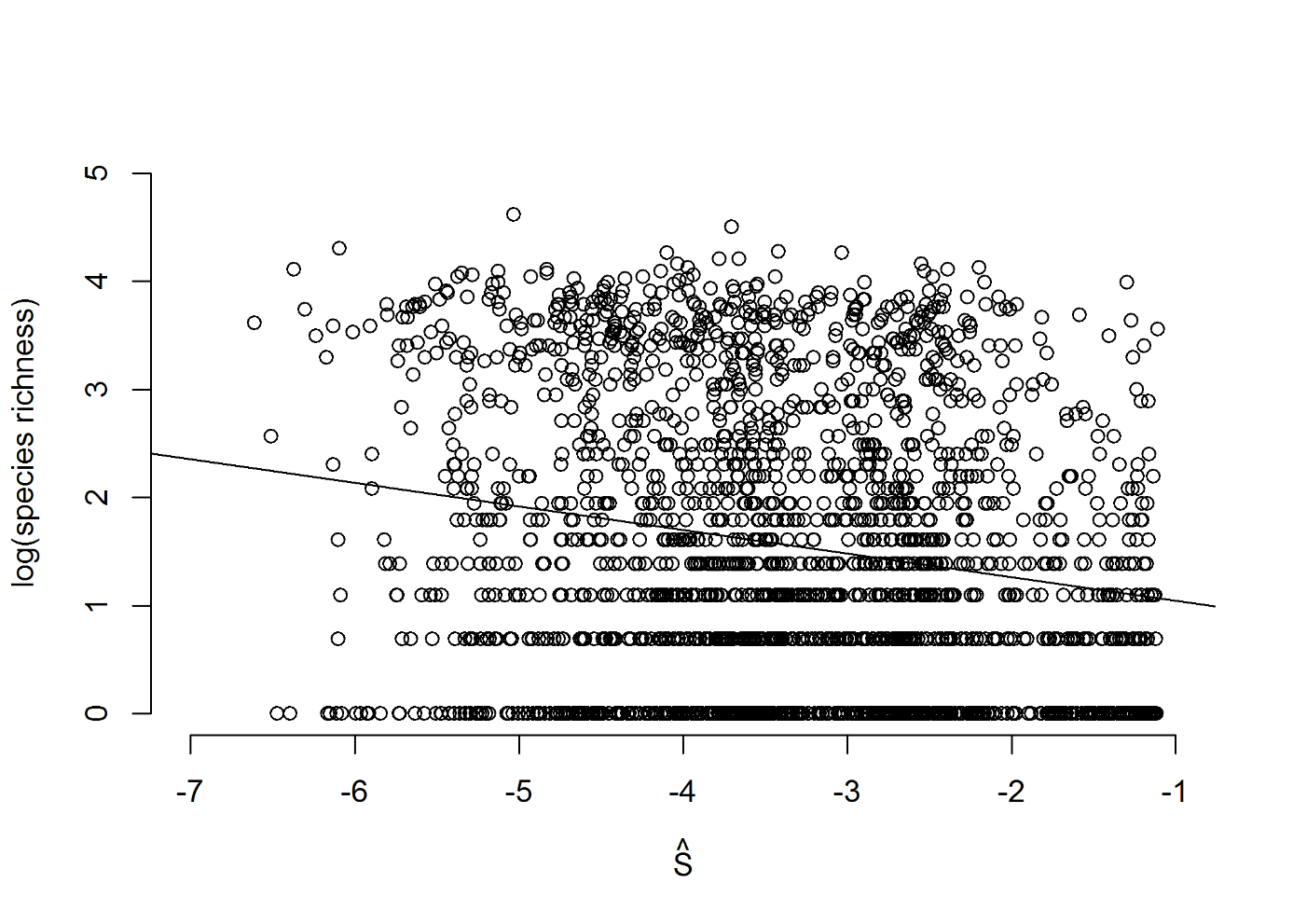
**

**Figure A.1** A scatterplot of log(species richness) versus the predictive mean of $S(x)$ in the LGCP model.

**
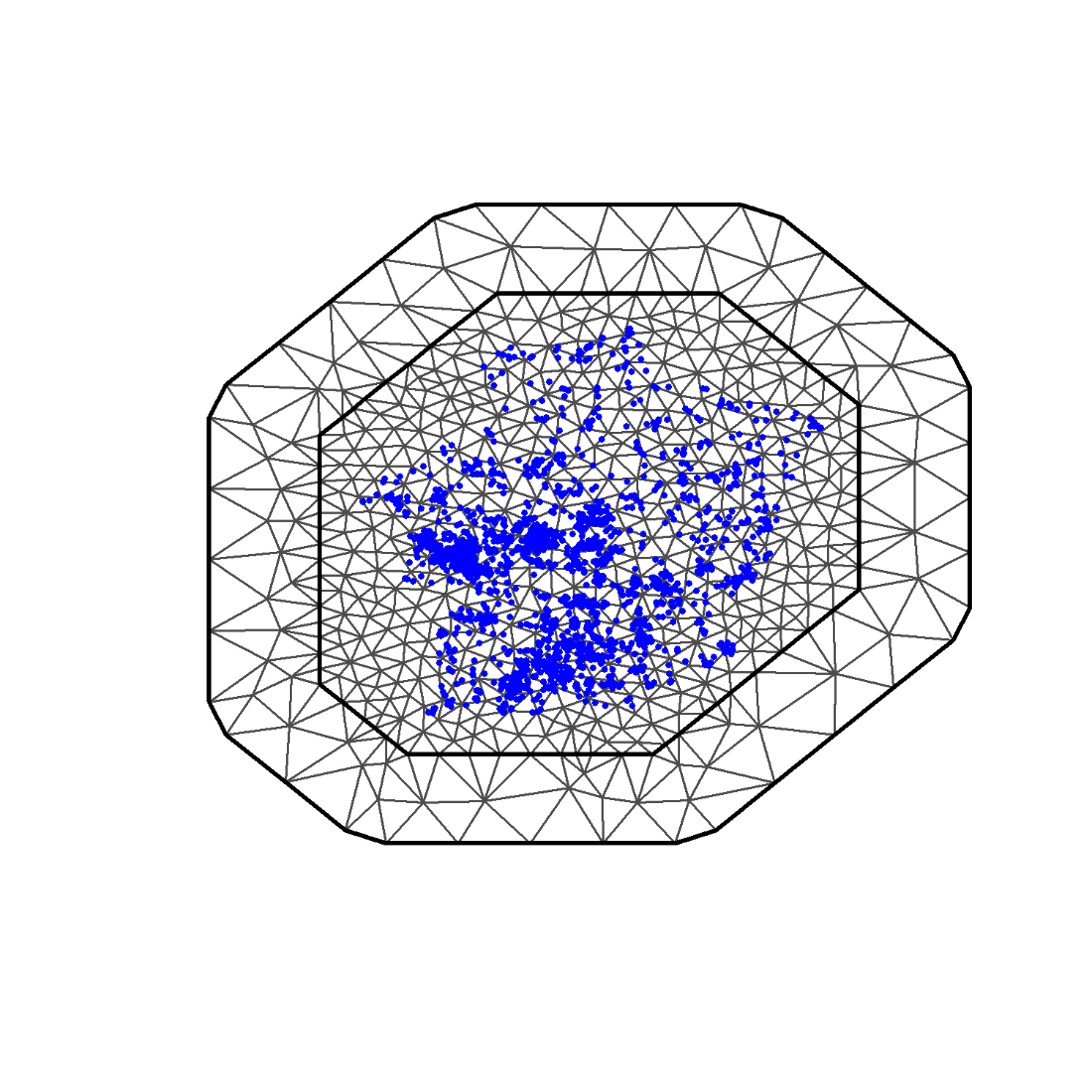
**

**Figure A.2** Triangulation mesh of Limburg, used for SPDE calculations. Blue dots represent observed sampling locations, with random jitter.
